# Highland cattle and *Radix labiata*, the hosts of *Fascioloides magna*

**DOI:** 10.1186/1746-6148-10-41

**Published:** 2014-02-11

**Authors:** Roman Leontovyč, Monika Košťáková, Veronika Siegelová, Klára Melounová, Jan Pankrác, Kristýna Vrbová, Petr Horák, Martin Kašný

**Affiliations:** 1 Faculty of Science, Department of Parasitology, Charles University in Prague, Viničná 7, Prague 128 44, Czech Republic; 2 Faculty of Science, Department of Botany and Zoology, Masaryk University, Kotlářská 2, Brno 611 37, Czech Republic

**Keywords:** *Fascioloides magna*, *Fasciola hepatica*, *Radix labiata*, *Galba truncatula*, Highland cattle, Molecular determination, ITS2, Histology, Pseudocyst

## Abstract

**Background:**

*Fascioloides magna* is a pathogenic fluke introduced to Europe ca 140 years ago. As it is spreading over the continent, new intermediate and definitive hosts might be involved in transmission of the parasite. In Europe, several studies reported potential new intermediate snail hosts (*Radix* spp.) for *F. magna*, and also several cases of fascioloidosis of wild and domestic animals were published. However, the data based on molecular and histological analyses confirming these findings remained unreported. This study aims to refer to unique findings of *F. magna* in European snails and domestic animals (the first observation in the Czech Republic in the last 30 years) and demonstrate the use of molecular techniques in determination of *F. magna*.

**Results:**

Two snails of *R. labiata* naturally infected with *F. magna* were found; mature cercariae and daughter rediae were observed. Maturity of cercariae was checked by histological methods, however, their ability to encyst was not confirmed. Co-infection of *F. magna* and *Fasciola hepatica* in the liver of two highland cattle bulls was proved. Adult fasciolid flukes producing eggs were found in the liver pseudocysts (*F. magna*) and the bile ducts (*F. hepatica*). Identification of intermediate hosts, intramolluscan stages, adult flukes and eggs was performed by sequencing the ITS2 region. Connection of *F. magna* pseudocysts with the gut (via the bile ducts) was not confirmed by means of histological and coprological examinations.

**Conclusions:**

For the first time, *Radix labiata* was confirmed as the snail host for *F. magna* under natural conditions and, together with the finding of *F. magna* infection in cattle, we can expect further transmission of *F. magna* from wildlife to livestock in localities shared by these hosts.

## Background

*Fascioloides magna* (Bassi, 1875) (Fasciolidae; Digenea; Trematoda) is a large visceral parasite of ruminants originating from North America. Due to the international wild animal trade, *F. magna* was introduced to Europe in the later half of the 19^th^ century, probably with the white-tailed deer (*Odocoileus virginianus*) [1] and wapiti (*Cervus elaphus canadensis*) [2]; the parasite adapted to the local hosts (e.g. red deer, fallow deer) and started to spread. Recent reports from at least six countries in central Europe can be found [3-9].

Like some other digenean trematodes, the *F. magna* life cycle includes two hosts, aquatic snails as intermediate hosts and ruminants as definitive hosts. In North America as the place of *F. magna* origin, at least five snail species of the family Lymnaeidae producing cercariae being able to form metacercariae were confirmed as the intermediate hosts [10-13]. In Europe only two naturally infected species within the family Lymnaeidae were found – *Galba truncatula*[1,3,14] and *Radix peregra* (in the light of recent discoveries, the latter species was probably *Radix labiata*) [15,16]. Only *G. truncatula* was shown to serve as the natural intermediate host emitting cercariae [1]. Some other lymnaeid snails distributed in the Czech Republic were tested under laboratory conditions as potential intermediate hosts of *F. magna*. Chroustová et al. [17] obtained cercariae (and subsequently infective metacercariae) from experimentally infected *Stagnicola palustris*. Erhardová-Kotrlá [1] successfully infected *Radix peregra peregra* and *R. peregra ovata* (according to the current nomenclature probably *Radix labiata* and *Radix balthica*) [15,18], nevertheless, only mother rediae were observed. Experimentally infected *Lymnaea stagnalis* was able to produce a low number of mature cercariae. This was in contrast to a massive exposure of *L. stagnalis* to miracidia and, therefore, *L. stagnalis* was considered to be a less susceptible intermediate host of *F. magna*[19]. *Omphiscola glabra* and *Pseudosuccinea columella* can also serve as the intermediate hosts of *F. magna*[20]. Concerning the latter two snails, there is no report confirming the occurrence of *O. glabra* in the Czech Republic, but *P. columella* was observed in several biotopes [21]. Since *P. columella* is a common snail in European greenhouses adapting to the central European climatic conditions [22], potential risk of *F. magna* transmission via this snail can not be excluded [20].

A wide spectrum of mammals can be infected with *F. magna*; these animals can generally be divided into three groups with respect to compatibility of the host and the parasite, and pathological impact of the fluke [11,23,24]. Successful completion of the *F. magna* life cycle with eggs released via bile ducts to the host intestine is enabled in “specific definitive hosts” such as red deer (*Cervus elaphus*), fallow deer (*Dama dama*) and roe deer (*Capreolus capreolus*) [1,11]. Infection with *F. magna* is tolerated by red deer and fallow deer which can survive, usually with hardly distinguishable symptoms, severe infection by hundreds of flukes. On the other hand, for roe deer the infection with a few flukes (less than 10 individuals) can be lethal [1]. The second category – “non-specific definitive hosts (dead-end hosts)” - is represented e.g. by moose (*Alces alces*), lama (*Lama glama*) and cattle (*Bos taurus*); these animals only exceptionally contribute to spreading of *F. magna*; the adult flukes can produce eggs, but these are trapped in liver pseudocysts not connected to the bile duct system [25-27]. Pathological impact on the “dead-end hosts” can differ. Whereas Foreyt and Todd [23] and Conboy and Stromberg [28] did not observe any significant health complications in infected animals, Chroustová et al. [17] recorded a reduced weight gain of infected animals (150 g/day). The third category comprises “non-typical hosts (aberrant hosts)” which are sensitive to *F. magna* infection, e.g., domestic sheep (*Ovis aries*) and domestic goat (*Capra hircus*) [24,28]; the immature flukes migrate continuously throughout the host body, cause serious damage to various organs, and finally they attack the liver which often leads to death [11,23].

In this paper two unique field findings of *F. magna* are described: larval stages of *F. magna* in the intermediate host *R. labiata*, and adult worms in the “non-specific definitive host” *Bos primigenius* f. *taurus* (highland cattle); the latter case is the first report of fascioloidosis in the Czech Republic in the last 30 years. Moreover, we refer to a unique case of co-infection of the same individual definitive host by *F. magna* and *F. hepatica*.

## Methods

### Ethics statement

The animals involved in the study were slaughtered in accordance with the regulations and recommendations of the Ministry of Agriculture of the Czech Republic and European Committee (The Veterinary Act 166/1999, EC regulation n. 1099/2009). The samples of the livers were examined by veterinary inspection and with the agreement of the farmers were forwarded to the team members at the Department of Parasitology, Faculty of Science, Charles University in Prague.

### Intermediate host (snail) sampling and examination

In June 2011 and July 2012, *Radix* spp. snails were collected at a locality with previous findings of *F. magna* – Sedliště game reserve [16] (GPS: 49°32′30.822″N 13°38′39.491″E). In addition, *G. truncatula* snails were sampled at the same locality for estimating the prevalence of *F. magna* in the snail population (see Table 1 for the number of collected snails).

**Table 1:** **Examination of *Radix *** **and *Galba *** **snails for *F. magna *** **larvae**

**Snail species**	**Year**	**No. examined**	**No. infected**	**Prevalence (%)**	**Developmental stage found**
*R. labiata*	2011	155	1	0.65	rediae, cercariae
	2012	970	1	0.10	rediae
	total	1125	2	0.18	
*G. truncatula*	2011	15	15	100	rediae, cercariae
	2012	35	16	49	rediae, cercariae
	total	50	31	64	

Snails were examined by dissection under stereomicroscope. Infected snails were washed and, together with cercariae and rediae, they were preserved in 96% ethanol for further molecular determination. In addition, hepatopancreases of infected snails were fixed in Bouin’s solution (Sigma-Aldrich) for further histological processing.

The species of snails were predominantly determined by molecular methods (see below). Morphological determination was based on morphometry/morphology of the shell and reproduction organs such as *bursa copulatrix* and its duct [15,29]. As morphological characters of reproduction organs can hardly be observed in infected individuals due to their destruction by larval stages of trematodes [30], only uninfected individuals of *Radix* spp. from the same locality were chosen for morphological determination.

### Definitive host (cattle) sampling and examination

Adult flukes of *F. magna* and *F. hepatica* were obtained from dissected livers of naturally infected highland cattle from South Bohemia (Czech Republic). The worms were preserved in 96% ethanol and Bouin’s solution for molecular and histological processing, respectively. Some living individuals were incubated in RPMI 1640 medium (Sigma) at 37°C overnight to obtain freshly laid eggs. In addition, coprological examination using the cattle faeces sampled directly from the gut content was performed. After sedimentation/decantation of fecal samples [31], the obtained sediment was examined under microscope and the egg-positive sediment samples were preserved in 96% ethanol for further molecular determination.

### Molecular determination

#### Snails

DNA was isolated from mechanically homogenized tissue using QIAamp® DNA Mini Kit, (QIAGEN). DNA concentration was measured by spectrophotometer ND-1000 (NanoDrop®) and then the samples were stored at -20°C. PCR was carried out in total volume of 25 μl using PPP Master Mix (Top-Bio), 50 ng of snail template DNA and 1 μl of 10 μM forward primer 5′ - TGT GTC GAT GAA GAA CGC AG – 3′ and reverse primer 5′ - TTC TAT GCT TAA ATT CAG GGG - 3′ specific for 502 bp ITS2 region [32].

#### Flukes

DNA was isolated from mechanically homogenized adult flukes (as described above for snail samples). Gene specific primers used for amplification of *F. magna* ITS2 region (152 bp) were designed according to Kráľová-Hromadová et al. [33] and Bazsalovicsová et al. [4] (forward 5′-ACCAGTTATCGTTGTGTTG-3′ and reverse 5′-CCGTCTTTAAACAACAG-3′).

#### Eggs

DNA was isolated using QIAamp® DNA Stool Mini Kit (QIAGEN) from *F. magna* eggs obtained after incubation of living worms, or from sediments of fecal samples. The eggs were homogenized in Bead-Beater (BioSpec) [34]. The same primers and procedure as for the adults were used (see above).

PCR was performed in C1000™ Thermal Cycler (Bio-Rad) with the following temperature profiles: 94°C 5 min, 30x (94°C, 30 sec; 63°C, 30 sec; 72°C, 2 min), 72°C, 10 min, 4°C hold. The obtained PCR products were examined by DNA electrophoresis (1.5% agarose gel with SYBR® Green I, Invitrogen); the gel with amplified DNA was cut out and purified using MinElute Gel Extraction Kit (QIAGEN) and sequenced by using the PCR primers and 3130xl Genetic Analyzer (Applied Biosystems).

All the obtained sequences were adjusted using DNA Star-Lasergene Core Suite software tool and compared with NCBI database using BLAST (Basic Local Alignment Search Tool).

### Histology

#### Intermediate hosts

Hepatopancreas of infected snails was fixed in Bouin’s solution (Sigma) and subsequently embedded in JB-4 Plus resin (Polysciences, Inc.). Sections (4 μm thick) were stained with hematoxylin-eosin.

#### Definitive hosts

After macroscopic examination of the host liver, the tissue samples with pathological changes were fixed in 4% buffered formaldehyde (for hematoxylin-eosin staining) or Bouin’s solution (for Gomori trichrome staining) and embedded in paraffin according to standard procedures. Sections (5 μm thick) were stained and examined under microscope (Olympus BX50).

## Results

### Examination of snails and determination of *F. magna* larval stages

In total, 1155 snails of the genus *Radix* were examined for fasciolid larval stages; two snails were infected and the prevalence was 0.18% (Table 1). Fifty individuals of *G. truncatula* from the same locality showed 64% prevalence. Molecular identification of rediae and mature cercariae showed that in both types of hosts the larvae belong to *F. magna*.

**Figure 1:**
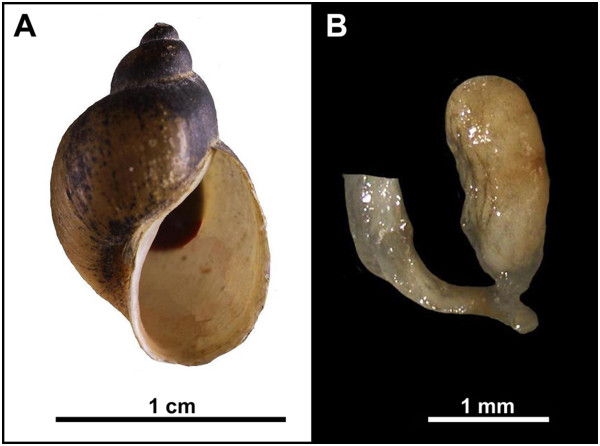
**Morphology of *Radix labiata*, shell (A) and bursa copulatrix (B).**

*Radix* snails infected with *F. magna* were determined as *R. labiata*. In addition, morphological characters corresponded to previous descriptions of *R. labiata*[15,18,29] (Figure 1).

### Histology of *F. magna* larval stages and snails

**Figure 2:**
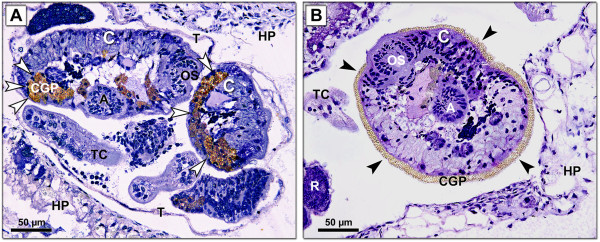
**Section of *Radix labiata *hepatopancreas with *Fascioloides magna *rediae and immature (A), mature (B) cercariae.** Stained with hematoxylin and eosin. HP, hepatopancreas tissue; C, cercaria; R, redia; T, tegument of redia; A, acetabulum; OS, oral sucker; TC, tail of cercaria; CGP, cystogenous gland products. White arrowheads point to cercarialcystogenous glands. Black arrowheads point to cercarial cystogenous products released from the glands.

Histological evaluation of the infected snail hepatopancreas of *R. labiata* revealed daughter rediae with developing cercariae, and also free mature cercariae covered by a thick layer of tegument and possessing cystogenous glands full of products (Figure 2A).

### Examination of definitive hosts and determination of *F. magna*/*F. hepatica* adults/eggs

**Figure 3:**
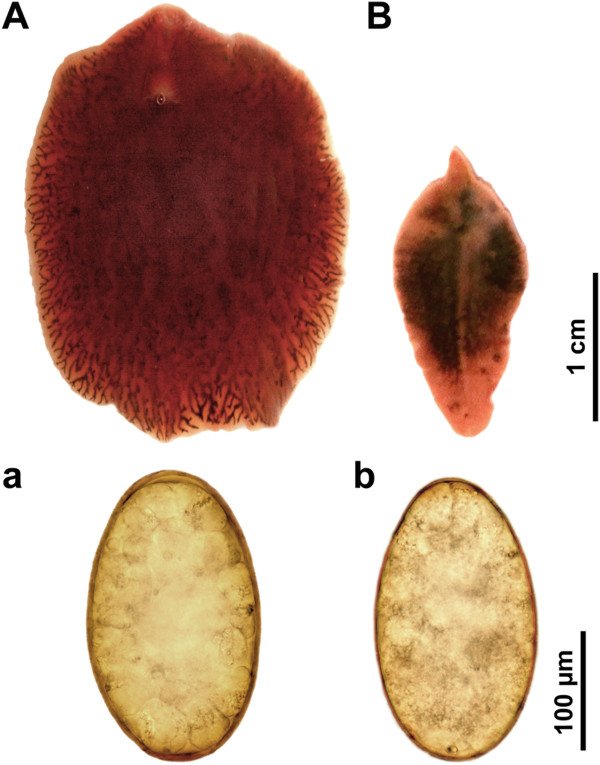
***Fascioloides magna *** **(A, a) and *Fasciola hepatica *** **(B, b), adults and eggs.**

**Figure 4:**
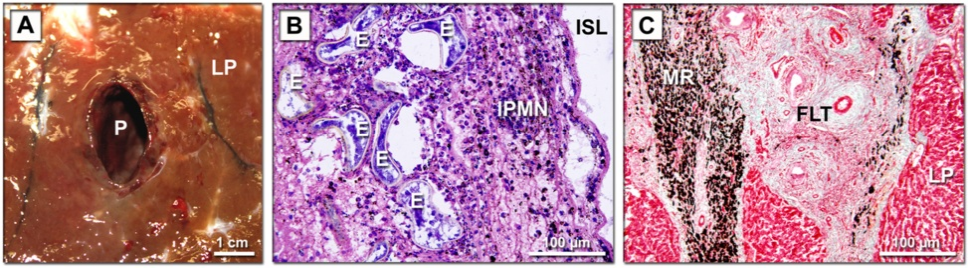
**Pathological changes in bovine liver caused by *F. magna *infection. A**, macroscopic photo of dissected liver tissue; **B**, microscopic photo – section of lesion with eggs and inflammatory infiltrate of polymorphonuclear leukocytes, stained with hematoxylin and eosin; **C**, microscopic photo – section of fibrotic portobiliar space and adjacent liver parenchyma, stained with green Gomori trichrome. P, pseudocyst; LP, liver parenchyma; E, egg of *F. magna*; IPMN, infiltrate of polymorphonuclear leukocytes; ISL, inner space of lesion; MR, migratory route through parenchyma with pigment deposits caused by *F. magna* juvenile worms; FLT, fibrotic liver tissue.

In January and September 2012 four three-years old bulls of the highland cattle were slaughtered in the slaughterhouse in South Bohemia. The dissected livers exhibited pathological changes showing the infection by fasciolid flukes - *F. hepatica* and *F. magna* (Figures 3, 4 and Table 2). The infected animals were gaunt and in bad physical condition.

**Table 2:** **Examination of livers from slaughtered highland cattle for *F. magna *** **adults**

		***F*. *hepatica***	***F*. *magna***	***F*. *magna (dead worms)***
January 2012	Bull 1	130	5	0
	Bull 2	187	0	0
September 2012	Bull 3	60	0	0
	Bull 4	50	2	5

#### Fasciola hepatica

All adult worms were found in the bile ducts with thickened and calcified walls. Beige mature flukes had maximum size of ca 3 cm and leaf-shaped body with typical oral cone and “shoulders” (Figure 3). After overnight incubation (37°C) in RPMI 1640, thousands of eggs were found and collected. Miracidia hatched after 16 days of incubation (25°C). Sequential analyses confirmed determination of both *F. hepatica* adults in the bile ducts and the eggs of *F. hepatica* in the faeces.

#### Fascioloides magna

Surface of the infected liver exhibited dark pigmentation associated with migration of juvenile flukes (the same dark lesions were recognized also in the case of *F. hepatica* infection). All individuals were localized in liver pseudocysts filled by a dark suspension of pigment, metabolites and eggs. The flukes had oval shape and reddish brown color. Length of the worms ranged from 3 cm to 5 cm. Pseudocysts with dead and particularly digested individuals were also observed. During overnight incubation (37°C) in RPMI 1640, the isolated mature living flukes produced eggs (several times less than *F. hepatica*); however, these eggs appeared unable to develop - after 16 days of incubation at 25°C no miracidia were observed. Sequential analyses confirmed the adult worms as *F. magna*. No eggs of *F. magna* were found in faeces.

## Discussion

Two *Radix* sp. snails naturally infected with fasciolid flukes were found at the locality Sedliště (south Bohemia – Czech Republic), and determined as *R. labiata* according to the sequence of ITS2 region; daughter rediae and mature cercariae were observed in the snail bodies. This is the first report on *Radix* sp. snails naturally infected with *F. magna* when morphological as well as molecular identifications have been performed. Faltýnková et al. [16] referred to a natural infection of *R. peregra*^a^ with *F. magna*, but the snail and the parasite determination was based on morphological characters only, and molecular confirmation was missing. Faltýnková et al. [16] collected the snails at the same locality (Sedliště game reserve) and, therefore, it is likely those snails were also *R. labiata* as investigated in our study. Moreover, the locality represents a typical biotope for *R. labiata*[35,36]. As the snails of the genus *Radix* occurring at the same locality can be infected with *F. magna* or *F. hepatica*[16,37], and their intramolluscan stages are morphologically indistinguishable, molecular determination needs to be performed to reach correct results [38]. The snails harboring both flukes were not found at the locality Sedliště. Recently, Huňová et al. [29] performed the first successful experimental infection of *R. labiata* with *F. magna*, followed by molecular identification of the snail and the parasite.

The prevalence of *F. magna*-infected *R. labiata* in Sedliště was 0.18% (similarly low prevalence from the same locality has been reported by Faltýnková et al. [16]), and the prevalence of infected *G. truncatula* snails was 64% (100% in 2011); such prevalence rate is quite unusual (e.g. [14]) and it indicates a high contamination of the locality by *F. magna* eggs.

Histological evaluation of hepatopancreas from naturally infected snails revealed freely moving cercariae with released cystogenous gland products on their surface (Figure 2). This indicates incorporation of a preparatory phase before encystation of cercariae in the outer environment [39]. However, no cercariae were able to encyst, probably because they were obtained artificially by dissection. Similar results were obtained by Erhardová-Kotrlá [1], Faltýnková et al. [16] and Huňová et al. [29] in experimental infections of *R. labiata* with *F. magna*^b^*.*

Two species of fasciolid flukes were found in the livers of two bulls of highland cattle; a typical localization of the flukes - bile ducts (*F. hepatica*) and fibrous pseudocysts (*F. magna*) - was observed. Morphological characters served for parasite determination, and identity of both species was subsequently verified by molecular methods*.* In the past, only one report on co-localized fasciolid flukes in one liver of cattle was published [25]. Coprological examination performed in our study revealed a high number of fasciolid eggs which were indistinguishable according to their morphology [1,40]. By applying molecular methods only the eggs of *F. hepatica* were determined. This finding corresponds to the literary data on pseudocysts that in the case of “non-specific definitive hosts (dead-end host)” are not connected via the bile ducts with the host gut, and the eggs accumulate in pseudocysts [23,28]. However, some exceptions may occur, e.g., Erhardová-Kotrlá [1] experimentally infected cattle and recorded eggs in the faecal samples, although in a low number. Balbo et al [9] and Foreyt and Todd [41] noticed a low number of eggs of *F. magna* in the faeces of naturally infected cattle. Foreyt and Todd [41] believed that the eggs may appear due to rupture of pseudocyst. Absence of eggs in the faeces of “non-specific definitive hosts (dead-end host)” complicates intravital diagnosis: it is impossible to apply routine coprological examination and, therefore, serological or other techniques should be used as an alternative.

Pathological impact of *F. magna* infection on cattle is questionable. Conboy and Stromberg [28] focused on analysis of blood samples. In addition, liver enzyme activities were monitored. These enzymes are probably a sensitive marker of liver damage by *F. hepatica*[42-44], however, in case of *F. magna* no significant difference between infected and uninfected individuals was recorded. Moreover, the infection (contrary to *F. hepatica* infection) did not affect the weight gain [45]. Besides cattle, also guinea pigs served for experimental infections. Although all the infected animals died and the pathological impact was evident, most of the monitored parameters were comparable to the control [28]. This implies that fascioloidosis is a serious disease with hardly observable diagnostic markers. In our study both bulls were in bad condition (e.g., chronic cachexy was mentioned in the veterinary report). We can speculate that it was probably due to *F. hepatica* infection, because in the liver of each bull at least 130 *F. hepatica* adult worms were found, whereas the number of *F. magna* adults was up to 10 individual flukes.

The infected bulls came from a South Bohemian biofarm with restricted therapeutic intervention and ideal natural condition for transmission of *F. magna*, e.g., wet pastures, natural streamlet and no effective fences. Erhardová-Kotrlá [1] carried out detailed research in the same locality and marked this locality as *F. magna* free after 6 cattle postmortem examinations and 282 examinations of the red deer faecal samples. Therefore, our results indicate that *F. magna* can spread in the Czech Republic.

## Conclusion

Two unique records of snail intermediate hosts (*R. labiata*) and mammalian definitive hosts (bulls of highland cattle) naturally infected with *F. magna* are reported in our study. Molecular techniques were used to confirm host/parasite determination; on this basis the adults of *F. magna* were discriminated from *F. hepatica* presented in the same liver. In addition, species identity of the morphologically uniform fasciolid eggs was proved. The prevalence of *F. magna* in *R. labiata* was comparable to some findings of *F. magna* in *G. truncatula* and, therefore, *R. labiata* might represent a potential vector of *F. magna* in localities ecologically unsuitable for *G. truncatula* (e.g. localities with acid soils). Ability of the parasite to be transmitted from wildlife to livestock represents a serious risk, especially for farmers breeding the animals less tolerant to *F. magna* infection (e.g., goats and sheep), and those animals whose are for some reason (e.g. ecological farming) under restrictions in terms of anthelmintic treatment. Therefore, the danger of *F. magna* transmission should be taken into account in farm management.

## Endnotes

^*a*^*R. peregra* is the taxonomic name which has generally been used by Czech malacologists. According to the results of generally accepted phylogenic analyses, *R. peregra* is synonymous with *R. labiata* which represents a valid taxon. In Western Europe the name of *R. peregra* was also used, but for different species currently named as *R. balthica*, see Schniebs et al. [15,18].

^b^Metacercariae of *F. magna* were found on the wall of Petri dish after dissection of experimentally infected *R. labiata* (Pankrác 2013, unpublished), but experimental infection of a definitive host was not performed.

## Competing interests

The authors declare that they have no competing interests.

## Authors’ contributions

RL - designed the study, participated in field collections, carried out morphological and molecular identification of snails and parasites, wrote the main parts of the manuscript; MKo - participated in field collections, carried out the analyses of histological samples; VS, KM – helped with molecular identification of snails and parasites; JP, KV - participated in field collections, helped with examination of snails; PH - helped with writing of the manuscript and interpretation of the data; MKa - designed the study, participated in field collections, coordinated laboratory work, helped with writing. All authors have read and approved the final manuscript.
